# Authors’ reply to: Letter to the Editor in response to ‘Parental attachment and depressive symptoms in pregnancies complicated by twin‐twin transfusion syndrome: a cohort study’

**DOI:** 10.1186/s12884-021-03687-8

**Published:** 2021-03-22

**Authors:** Fiona L. Mackie, R. Katie Morris, Mark D. Kilby

**Affiliations:** 1 Department of Obstetrics and Gynaecology, Worcestershire Acute NHS Trust, Royal Worcestershire Hospital, Charles Hastings Way, WR5 1DD Worcester, UK; 2 Institute of Applied Health Research, University of Birmingham, West Midlands Edgbaston, UK; 3 Fetal Medicine Centre, Birmingham Women’s and Children’s NHS Foundation Trust, Birmingham Women’s Hospital, Mindelsohn Way, Edgbaston, UK; 4 Institute of Metabolism and Systems Research, College of Medical and Dental Sciences, University of Birmingham, Birmingham, UK; 5 Centre for Women’s and Children’s Health, Birmingham Health Partners, Birmingham, UK

**Keywords:** Twin twin transfusion syndrome, Parental attachment, Anxiety, Fetoscopic laser ablation, Mental health

## Abstract

In this correspondence we thank the authors for highlighting the importance of our work, and agree with the limitations they have raised regarding performing this study.

## Main text

**Dear Editor**

We thank Rameh et al. [1] for highlighting the importance of our work [2]. The limitations of our study, as stated by Rameh et al. are mentioned in our published article. The need to interpret our findings of the post-ablation and postnatal questionnaires with caution due to the low numbers was highlighted in our article. We agree that future research should explore the additional potential associations with anxiety, coping styles in times of stress, the parent’s own attachment style, and romantic attachment to their partner on parental attachment. Ideally these associations should be explored with validated assessment tools. Interviews with parents would give additional useful information and may improve follow-up data collection. Additionally future studies should be performed in larger cohorts which would require collaboration between treatment centres. We hope that our study will improve patient care by increasing health care professional and patient awareness of the need to risk assess and screen mothers and fathers going through a pregnancy complicated by twin-twin transfusion syndrome for mental health problems, and consequently enabling additional psychological support where needed.

## Data Availability

Not applicable.
